# *Triatoma sordida* (Hemiptera, Triatominae) from La Paz, Bolivia: an incipient species or an intraspecific chromosomal polymorphism?

**DOI:** 10.1186/s13071-021-04988-9

**Published:** 2021-10-27

**Authors:** Fernanda Fernandez Madeira, Luiza Maria Grzyb Delgado, Isadora de Freitas Bittinelli, Jader de Oliveira, Amanda Ravazi, Yago Visinho dos Reis, Ana Beatriz Bortolozo de Oliveira, Daniel Cesaretto Cristal, Cleber Galvão, Maria Tercília Vilela de Azeredo-Oliveira, João Aristeu da Rosa, Kaio Cesar Chaboli Alevi

**Affiliations:** 1 Laboratório de Biologia Celular, Universidade Estadual Paulista “Júlio de Mesquita Filho” (UNESP), Instituto de Biociências, Letras e Ciências Exatas, Rua Cristóvão Colombo, 2265, São José do Rio Preto, SP 15054-000 Brasil; 2 Universidade Estadual Paulista “Júlio de Mesquita Filho” (UNESP), Instituto de Biociências Rua Dr. Antônio Celso Wagner Zanin, 250, Distrito de Rubião Júnior, Botucatu, SP 18618-689 Brasil; 3 Laboratório de Parasitologia, Universidade Estadual Paulista “Júlio de Mesquita Filho” (UNESP), Faculdade de Ciências Farmacêuticas, Rodovia Araraquara-Jaú km 1, Araraquara, SP 14801-902 Brasil; 4 Laboratório de Entomologia em Saúde Pública, Departamento de Epidemiologia, Faculdade de Saúde Pública, Universidade de São Paulo, Av. Dr. Arnaldo 715, São Paulo, SP Brasil; 5 Laboratório Nacional e Internacional de Referência em Taxonomia de Triatomíneos, Instituto Oswaldo Cruz (FIOCRUZ), Av. Brasil 4365, Pavilhão Rocha Lima, sala 505, Rio de Janeiro, RJ 21040-360 Brasil

**Keywords:** Chagas disease vector, Triatomines, Experimental crosses, Genetic distance

## Abstract

**Background:**

*Triatoma sordida* is one of the main Chagas disease vectors in Brazil. In addition to Brazil, this species has already been reported in Bolivia, Argentina, Paraguay, and Uruguay. It is hypothesized that the insects currently identified as *T. sordida* are a species subcomplex formed by three cytotypes (*T. sordida *sensu stricto [s.s.], *T. sordida* La Paz, and *T. sordida* Argentina). With the recent description of *T. rosai* from the Argentinean specimens, it became necessary to assess the taxonomic status of *T. sordida* from La Paz, Bolivia, since it was suggested that it may represent a new species, which has taxonomic, evolutionary, and epidemiological implications. Based on the above, we carried out molecular and experimental crossover studies to assess the specific status of *T. sordida* La Paz.

**Methods:**

To evaluate the pre- and postzygotic barriers between *T. sordida* La Paz and *T. sordida *s.s., experimental crosses and intercrosses between F1 hybrids and between F2 hybrids were conducted. In addition, cytogenetic analyses of the F1 and F2 hybrids were applied with an emphasis on the degree of pairing between the homeologous chromosomes, and morphological analyses of the male gonads were performed to evaluate the presence of gonadal dysgenesis. Lastly, the genetic distance between *T. sordida* La Paz and *T. sordida *s.s. was calculated for the *CYTB*, *ND1*, and *ITS1* genes.

**Results:**

Regardless of the gene used, *T. sordida* La Paz showed low genetic distance compared to *T. sordida *s.s. (below 2%). Experimental crosses resulted in offspring for both directions, demonstrating that there are no prezygotic barriers installed between these allopatric populations. Furthermore, postzygotic barriers were not observed either (since the F1 × F1 and F2 × F2 intercrosses resulted in viable offspring). Morphological and cytogenetic analyses of the male gonads of the F1 and F2 offspring demonstrated that the testes were not atrophied and did not show chromosome pairing errors.

**Conclusion:**

Based on the low genetic distance (which configures intraspecific variation), associated with the absence of prezygotic and postzygotic reproductive barriers, we confirm that *T. sordida* La Paz represents only a chromosomal polymorphism of *T. sordida *s.s.

**Graphical abstract:**

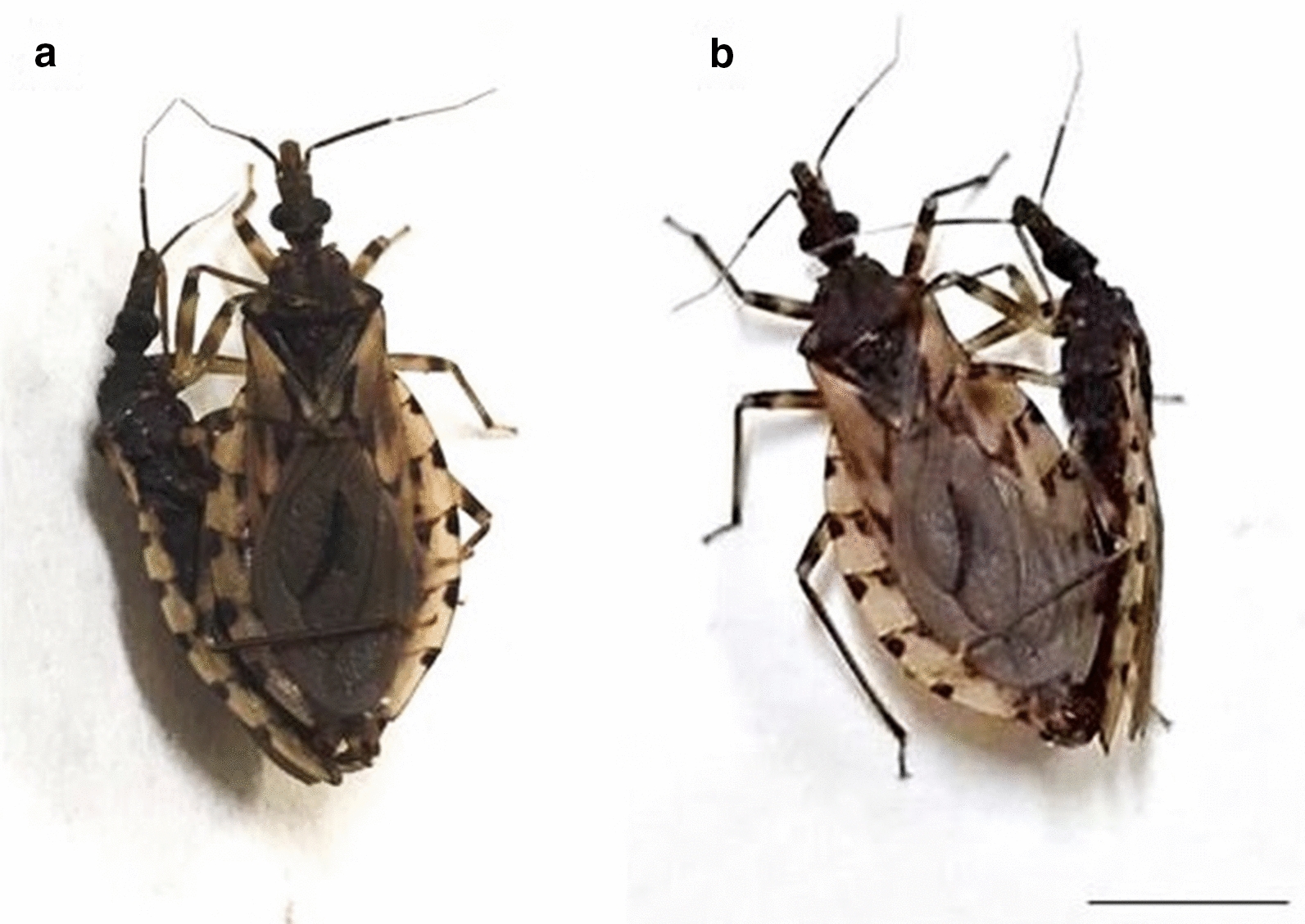

## Background

Triatomines are hematophagous insects of great importance to public health, since they are considered the main form of transmission of the protozoan *Trypanosoma cruzi* (Chagas, 1909) (Kinetoplastida, Trypanosomatidae), the etiological agent of Chagas disease [1]. This disease (also known as American trypanosomiasis) is a neglected disease that affects about eight million people worldwide and results in 10,000 deaths per year [1]. These insects defecate or urinate during or after hematophagy and, once infected by *T. cruzi*, release the infective form of the parasite in feces/urine [1], which may lead to contamination of the host.

The subfamily Triatominae (Hemiptera, Reduviidae) is composed of 156 species, grouped into 18 genera and five tribes [2–4]. All species are considered potential vectors of Chagas disease, although the genera *Triatoma* Laporte, 1832, *Rhodnius* Stål, 1859, and *Panstrongylus* Berg, 1879, are considered the most important from an epidemiological point of view [5, 6].

*Triatoma sordida* (Stål, 1859) is one of the main Chagas disease vectors in Brazil, being the most commonly captured species in peridomestic Brazilian environments [5, 7], where it has already been reported to be naturally infected by *T. cruzi* [8]. In addition to Brazil, this species has been reported in Bolivia, Argentina, Paraguay, and Uruguay, in the Atlantic Forest, Caatinga, Cerrado, Chaco, and Pantanal biomes [3, 5, 9]. It is hypothesized that the insects currently identified as *T. sordida* could actually be a species subcomplex formed by three cytotypes (cytotype 1 determined as *T. sordida *sensu stricto [s.s.], cytotype 2 determined as *T. sordida* La Paz, and cytotype 3 determined as *T. sordida* Argentina) [9], which resulted in the recent description of *T. rosai* Alevi et al., 2020, from the Argentinean specimens (excluding Argentina in the geographic distribution of *T. sordida*) [3].

It was long believed that the diversification events of the different *T. sordida* cytotypes were based on cryptic speciation [9]. However, comparative morphological and morphometric studies between *T. rosai* and *T. sordida* (Brazil, Bolivia, and Paraguay), as well as between *T. sordida* from different countries, found phenotypic differences, demonstrating that it is not cryptic speciation [10]. With the recent description of *T. rosai* (from *T. sordida* cytotypes from Argentina), it became necessary to assess the taxonomic status of *T. sordida* from La Paz, Bolivia (referred to as *T. sordida* La Paz), since Panzera et al. [9] suggested that this cytotype composed of domestic populations from La Paz, Bolivia, may represent a new species, which has taxonomic, evolutionary and, above all, epidemiological implications.

According to Panzera et al. [9], *T. sordida* La Paz represents a “chromosomal taxon,” but these authors do not rule out the possibility that *T. sordida* La Paz and *T. sordida *s.s. may be conspecific populations with different ribosomal gene locations. Morphological, morphometric [11], and phylogenetic [12] studies have pointed to this hypothesis as the most viable. Assessing the specific status of *T. sordida* La Paz also has epidemiological implications, since different species may have different vectorial capacity and vector competence, such as *T. sordida *s.s. and *T. rosai*, which have different infection rates [3, 13–17]. Entomo-epidemiological studies indicate different infection rates between *T. sordida* from Brazil (0.5–41.9%) and Bolivia (16.2%) [14–18]. Thus, based on the above, we carried out molecular and experimental crossover studies to assess the specific status of *T. sordida* La Paz.

## Methods

### Experimental crosses

Experimental reciprocal crosses were conducted between *T. sordida* La Paz (Apolo, La Paz, Bolivia, collected in peridomiciliary ecotopes) and *T. sordida *s.s. (Corumbá, Mato Grosso do Sul, Brazil, collected in peridomiciliary ecotopes). The insects used in the experiments came from colonies kept in the Triatominae insectary of the School of Pharmaceutical Sciences, São Paulo State University (UNESP), Araraquara, São Paulo, Brazil. The experimental crosses were conducted in the Triatominae insectary, according to the experiments of Correia et al. [19] and Mendonça et al. [20]: the insects were sexed as fifth-instar nymphs [21], and males and females were kept separate until they reached the adult stage to guarantee the virginity of the insects used in the crosses. For the experimental crosses, three couples from each set were placed in plastic jars (diameter 5 cm × height 10 cm) and kept at room temperature (average of 24 °C [22]) and 63% average relative humidity [22]. Weekly, the eggs were collected, counted, and separated in new jars to assess the hatch rate. Upon reaching the fifth instar (N5), again three pairs of first-generation nymphs (F1) resulting from each interspecific cross were separated to perform intercrosses (F1 × F1) (Table 1), using the same parameters described above and again reviewed. Finally, the eggs of these crosses were counted and separated, and from the second-generation nymphs (F2), new backcrosses (F2 × F2) (Table 1) were performed to assess the hatching rate of the third generation (F3).

**Table 1:** Experimental crosses performed between *T. sordida* La Paz and *T. sordida *s.s.

	Number of eggs				Egg fertility (%)
	C1	C2	C3	Total	
Crossing experiments					
*T. sordida* La Paz ♀ × *T. sordida *s.s. ♂	60	92	200	352	86
*T. sordida *s.s. ♀ × *T. sordida* La Paz ♂	64	68	225	357	85
Hybrid F1 ♀ × Hybrid F1 ♂^a^	79	274	–	353	85
Hybrid F1 ♀ × Hybrid F1 ♂^b^	189	108	–	297	93
Hybrid F2 ♀ × Hybrid F2 ♂^c^	200	139	–	339	64
Hybrid F2 ♀ × Hybrid F2 ♂^d^	122	150	–	272	67
Control experiments					
*T. sordida* La Paz ♀ × *T. sordida* La Paz ♂	132	164	35	331	53
*T. sordida *s.s. ♀ × *T. sordida *s.s. ♂	286	126	178	590	73

Hybrids of the crosses: ^a^between *T. sordida* La Paz ♀ × *T. sordida *s.s. ♂; ^b^between *T. sordida *s.s. ♀ × *T. sordida* La Paz ♂; ^c^between hybrids F1 from *T. sordida* La Paz ♀ × *T. sordida *s.s. ♂; ^d^between hybrids F1 from *T. sordida *s.s. ♀ × *T. sordida* La Paz ♂. C1, C2, and C3: replicates of experimental crosses, F1: first-generation, F2: second-generation

### Molecular analysis

For molecular analysis, the genomic DNA of five parental insects used in crosses (*T. sordida* Apolo, La Paz, Bolivia, and *T. sordida* Corumbá, Mato Grosso do Sul, Brazil, both collected in peridomiciliary ecotopes) was extracted from gonads using the DNeasy Blood and Tissue Kit (QIAGEN). Amplification of the fragments was performed by polymerase chain reaction (PCR), using primers targeting the *CYTB* [23], *ND1* [24], and *ITS1* genes [25] described in the literature. The amplified PCR products were visualized by electrophoresis in 1% agarose gel and later purified using the GFX PCR DNA and Gel Band Purification Kit (GE Healthcare) according to the manufacturer's instructions. Subsequently, this material was submitted for direct sequencing using an ABI 3730 DNA Analyzer (Applied Biosystems) sequencer, from the Human Genome and Stem Cell Research Center, University of São Paulo (USP), Brazil. The obtained sequences were manually edited using the BioEdit alignment editor v.7.0.5.3 and aligned using the ClustalW multiple sequence alignment tool. The genetic sequences are available on GenBank [access data: *T. sordida *s.s. *CYTB* (MZ700100), *ND1* (MZ700102), and *ITS1* genes (MZ648337); *T. sordida* La Paz *CYTB* (MZ700101), *ND1* (MZ700103), and *ITS1* genes (MZ648333)]. The genetic distance matrices between *T. sordida *s.s. and *T. sordida* La Paz for all genes were obtained with MEGA 7.0 software, using the Kimura-2-parameter distance model (K2P). Sequences available from GenBank of *T. brasiliensis* Neiva, 1911, for the *CYTB* (KC249239), *ND1* (AM980619), and *ITS1* genes (KJ125138) were used as outgroups.

### Morphology of the gonads and cytogenetic analysis

After the experimental crosses (F1 × F1 and F2 × F2) (Table 1), the F1 and F2 males were dissected (*n* = 5), the testes were removed and stored in methanol/acetic acid solution (3:1). Before cytogenetic analysis, the morphology of the male gonads from the F1 and F2 hybrid specimens (*n* = 5) was analyzed under a Leica MZ APO stereoscopic microscope and analyzed through the Motic Images Advanced 3.2 image analysis system to evaluate the presence of gonadal dysgenesis (which may be unilateral or bilateral) [26]. Posteriorly, slides were prepared by the cell crushing technique as described by Alevi et al. [27]), and cytogenetic analysis was applied to characterize the spermatogenesis, with emphasis on the degree of pairing between the homeologous chromosomes [20], using the lacto-acetic orcein technique [27, 28]. The slides were examined using a Jenaval light microscope (Zeiss), coupled to a digital camera and AxioVision LE 4.8 image analysis software. The images obtained were magnified by a factor of ×1000.

## Results and discussion

Extremely low genetic distance values were found between *T. sordida* La Paz and *T. sordida *s.s. (Table 2). These results could be associated with the absence of prezygotic [represented by the hatching of F1 hybrids (Table 1)] and postzygotic barriers [represented by the absence of mortality among F1 hybrids that showed normal development and reached adulthood (absence of hybrid inviability) (Table 1), by obtaining F2 hybrids, demonstrating that the F1 hybrids were fertile (Table 1) and that they did not present atrophied gonads (Fig. 2) and produced viable gametes (Fig. 3) (absence of hybrid sterility) and by high F3 hatching rate (Table 1) associated with 100% pairing (Fig. 3) (absence of hybrid collapse)] installed between these allopatric populations (Table 1). These data indicate that *T. sordida* La Paz represents only a chromosomal polymorphism of *T. sordida *s.s.

**Table 2:** Genetic distance between *T. sordida* La Paz and *T. sordida *s.s. for mitochondrial (*CYTB* and *ND1*) and nuclear (*ITS1*) genes

Genetic distance of *CYTB* gene			
	(1)	(2)	(3)
(1) *T. sordida *s.s.	–		
(2) *T. sordida* La Paz	0.018		
(3) *T. brasiliensis*	0.184	0.192	–

Genetic distance of *ND1* gene			
	(1)	(2)	(3)
(1) *T. sordida *s.s.	–		
(2) *T. sordida* La Paz	0.019		
(3) *T. brasiliensis*	0.098	0.089	–

Genetic distance of *ITS1* gene			
	(1)	(2)	(3)
(1) *T. sordida *s.s.	–		
(2) *T. sordida* La Paz	0.008		
(3) *T. brasiliensis*	0.253	0.244	–

Alevi et al. [3] and Belintani et al. [12] presented robust phylogenetic analyses using *T. sordida *s.s., *T. sordida* La Paz, and *T. rosai*. In both, *T. sordida* La Paz was recovered along with *T. sordida *s.s., and *T. rosai* was recovered as an independent strain. Morphological and morphometric analyses resulted in similar clusters as those above, since *T. sordida *s.s. and *T. sordida* La Paz showed characteristics similar to each other (that grouped them) and divergent from *T. rosai* [11].

Regardless of the gene used, *T. sordida* La Paz showed extremely low genetic distance compared to *T. sordida *s.s. (Table 2). Monteiro et al. [29] suggest that minimum interspecific distance in Triatominae should be greater than 2%. *Triatoma rosai* and *T. sordida *s.s., for example, have a distance greater than 8% (which confirmed the specific status of *T. rosai*) [3]. On the other hand, our results demonstrated that the greatest distance observed between *T. sordida* La Paz and *T. sordida *s.s. was 1.9% (Table 2), which suggests that these cytotypes represent only intraspecific chromosomal variation.

**Fig. 1:**
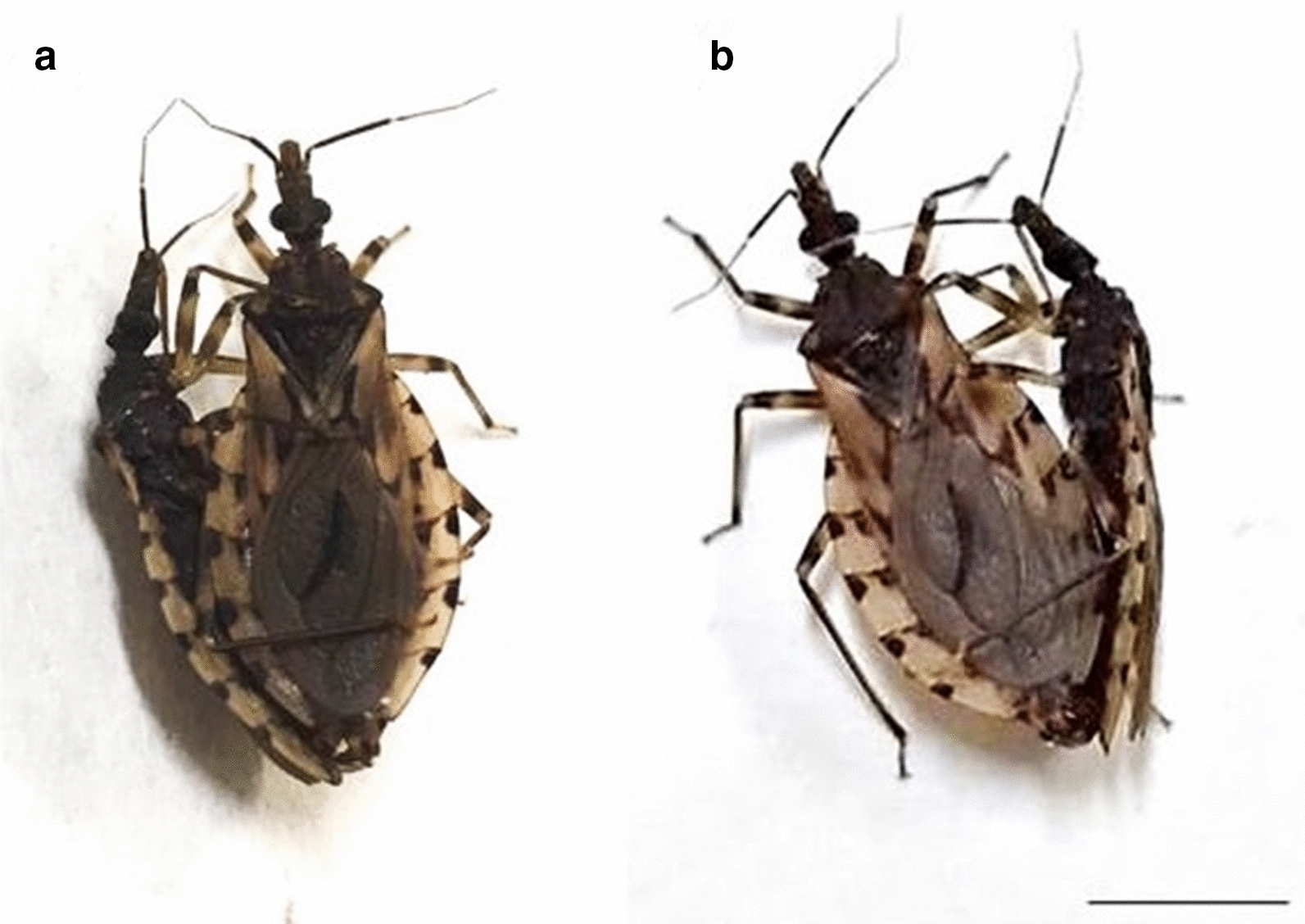
Experimental crosses between *T. sordida* La Paz and *T. sordida *s.s.: **a** *T. sordida *s.s. female (right) × *T. sordida* La Paz male (left); **b** *T. sordida* La Paz female (left) × *T. sordida *s.s. male (right). Bar: 1 cm

Experimental crosses (Fig. 1) resulted in offspring for both directions (Table 1), demonstrating that there are no prezygotic barriers installed between these allopatric populations. These results demonstrate that beyond the physical barriers that may make it impossible to find these insects together in nature (because they inhabit different countries in South America [9]), there are no prezygotic barriers installed between *T. sordida* La Paz and *T. sordida *s.s. Therefore, under laboratory conditions, these insects recognize each other (absence of ethological isolation) and copulate (absence of mechanical isolation), in addition to demonstrating gametic, genomic and, above all, zygotic (absence of gametic isolation) compatibility.

In triatomines, the detection of pre- and/or postzygotic barriers has helped in taxonomy and systematics, since evolutionarily more distant species have prezygotic barriers that prevent the formation of hybrids, while evolutionarily closely related species can produce hybrids that will decline (hybrid breakdown) by postzygotic barriers such as hybrid inviability, hybrid sterility, or hybrid collapse [30].

**Fig. 2:**
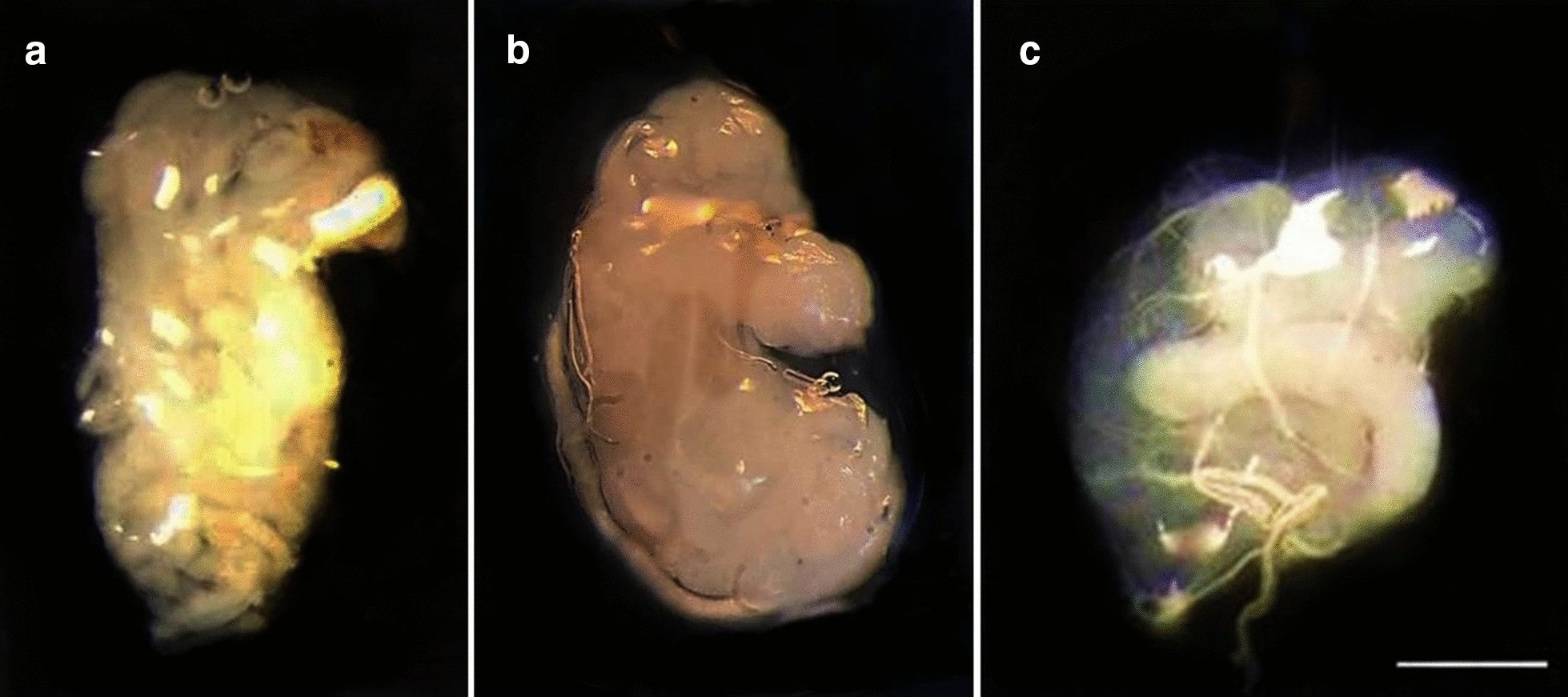
Non-atrophied testicle of hybrid of *T. sordida* La Paz and *T. sordida *s.s., demonstrating the absence of gonadal dysgenesis. **a** Testicle of *T. sordida* La Paz; **b** testicle of hybrid F1 of the crosses between *T. sordida* La Paz female × *T. sordida *s.s. male; **c** testicle of hybrid F2 of the crosses between *T. sordida* La Paz male × *T. sordida *s.s. female. Bar: 10 mm

**Fig. 3:**
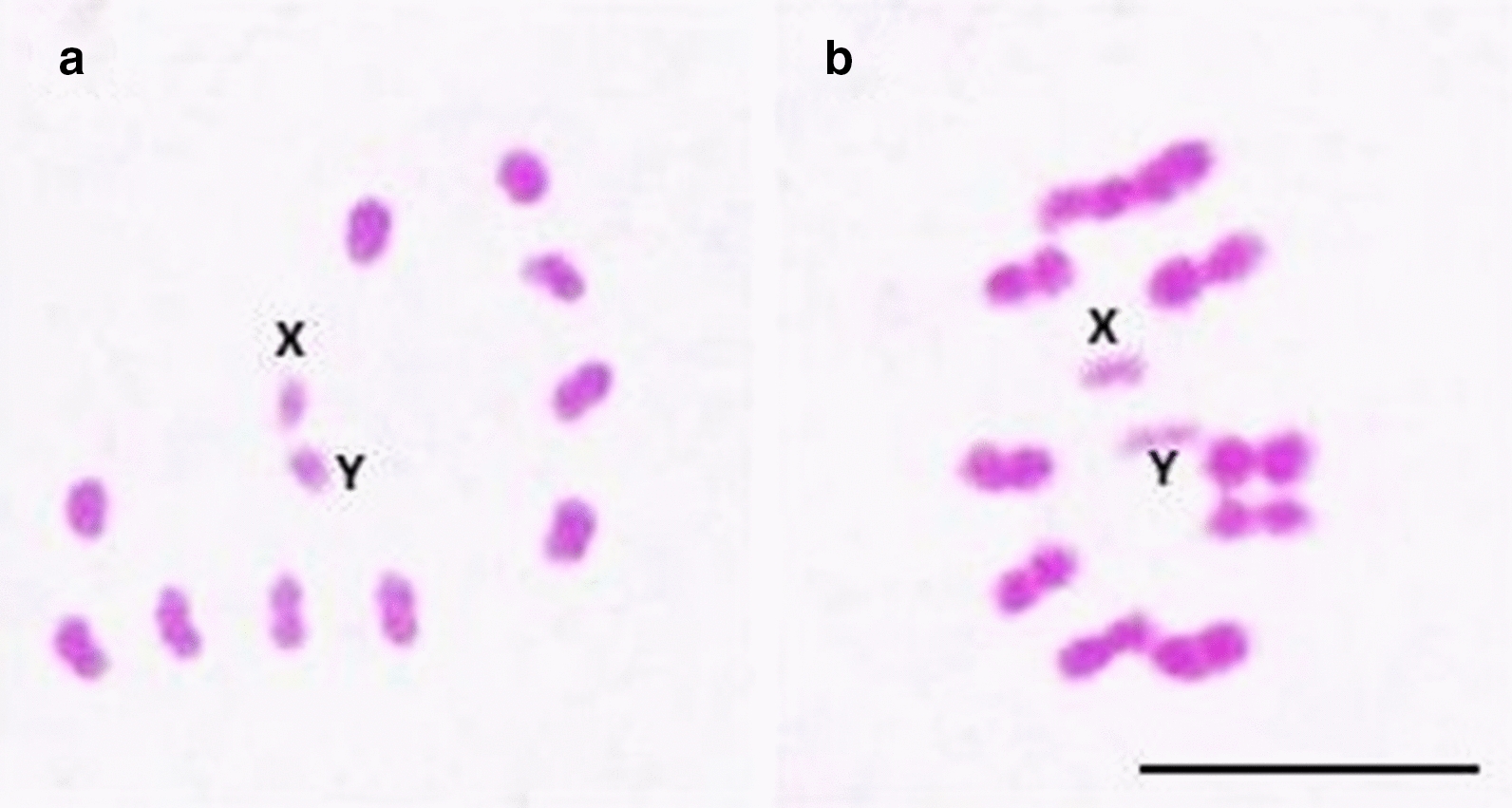
Meiotic metaphases of hybrids F1 resulting from the experimental crosses between *T. sordida* La Paz and *T. sordida *s.s. **a**, **b** Metaphases with correct pairing of the homeologous chromosomes. **a** Hybrid F1 of the crosses between *T. sordida* La Paz female × *T. sordida *s.s. male; **b** hybrid F1 of the crosses between *T. sordida* La Paz male × *T. sordida *s.s. female; X: sex chromosomes X, Y: sex chromosomes Y. Bar: 10 μm

From F1 hybrids that reached adulthood, confirming the absence of postzygotic barrier of hybrid inviability, intercrosses were performed (Table 1) and F2 hybrids were obtained for all combinations (Table 1), also demonstrating the absence of the postzygotic barrier of hybrid sterility. The unfeasibility of the hybrids is represented by the high mortality rate, associated with their low adaptive value, during the nymphal stages [3]. This postzygotic barrier was extremely important in confirming the specific status of *T. rosai* [3]. On the other hand, hybrid sterility, which can be represented by atrophy of the gonads (gonadal dysgenesis) [26] or by chromosomal pairing errors which lead to the formation of unviable gametes [31], was also not observed, since F2 hybrids hatched (Table 1), and morphological analyses of the male gonads of the F1 and F2 offspring demonstrated that the testes were not atrophied compared to the control group—absence of gonadal dysgenesis (Fig. 2). Furthermore, cytogenetic analyses of the degree of pairing between the homeologous chromosomes of the F1 and F2 hybrids demonstrated genomic compatibility between the two cytotypes, as all chromosomes were paired during metaphases (Fig. 3), resulting in the formation of viable gametes.

For triatomines, all cases of hybrid sterility were associated only with errors during meiosis [31, 32]. The degree of pairing between the homeologous chromosomes of F1 hybrids allows us to evaluate the evolutionary relationship between the species [33]: in general, species that are phylogenetically closer range from about 90 to 100% chromosomal pairing (as observed among species of the *T. brasiliensis* complex [20, 34] and *T. infestans* subcomplex [23]), while more distantly related species show several pairing errors (40% or more), as observed between *T. infestans* and *T. rubrovaria* (Blanchard, 1843) [31]. Panzera et al. [9] presented cytogenetic analyses of four possible hybrids of *T. sordida* from Bolivia (two from Apolo, La Paz, and two from Izozog, Santa Cruz) and observed 100% chromosomal pairing and variations in 45S rDNA probe markings (which led the authors to consider these specimens hybrids). Our results indicate that the insects analyzed were only chromosomal variants of *T. sordida *s.s.

Nascimento et al. [35] combined molecular data and experimental crosses to synonymize *R. taquarussuensis* da Rosa et al., 2017, with *R. neglectus* Lent, 1954. The authors noted that the hatch rates of F1 offspring were the same as for parental crosses (greater than 78%). Our results demonstrate that the hatching rate of the F1 and F2 offspring was greater than 80%, and that of the F3 offspring was greater than 60% (Table 1), both of which were higher than the hatching rate observed to control crosses between *T. sordida* La Paz (Table 1). These results, combined with the low genetic distance data, also indicate that *T. sordida* La Paz and *T. sordida *s.s. are the same species.

The hatching of viable hybrids up to F3 demonstrates the absence of a postzygotic barrier by hybrid collapse. Mendonça et al. [20] characterized this event for hybrids resulting from the crosses between *T. lenti* Sherlock & Serafim, 1967, and *T. sherlocki* Papa et al., 2002. The authors observed the absence of F3 for the cross between *T. lenti* female and *T. sherlocki* male and the presence of only two F3 nymphs for the cross between *T. lenti* male and *T. sherlocki* female, which died soon after hatching [20]. Furthermore, Mendonça et al. [20] analyzed the metaphases of F2 hybrids that were intercrossed and observed several chromosomal pairing errors (which resulted in the formation of nonviable gametes). The high F3 hatching rate associated with 100% pairing between the F2 hybrid chromosomes demonstrates that this evolutionary phenomenon is not occurring in hybrids of *T. sordida* La Paz and *T. sordida *s.s.

## Conclusion

Based on the low genetic distance (which configures intraspecific variation) associated with the absence of prezygotic and postzygotic reproductive barriers, we confirm that *T. sordida* La Paz represents only a chromosomal polymorphism of *T. sordida *s.s.

## Data Availability

The data supporting the conclusions of this article are included within the article.
